# miRNA-124 in Immune System and Immune Disorders

**DOI:** 10.3389/fimmu.2016.00406

**Published:** 2016-10-04

**Authors:** Zhen Qin, Peng-Yuan Wang, Ding-Feng Su, Xia Liu

**Affiliations:** ^1^Department of Pharmacology, School of Pharmacy, Second Military Medical University, Shanghai, China

**Keywords:** miRNA-124, biomarker, target, immunity, immune disorders

## Abstract

In recent years, miR-124 has emerged as a critical modulator of immunity and inflammation. Here, we summarize studies on the function and mechanism of miR-124 in the immune system and immunity-related diseases. They indicated that miR-124 exerts a crucial role in the development of immune system, regulation of immune responses, and inflammatory disorders. It is evident that miR-124 may serve as an informative diagnostic biomarker and therapeutic target in the future.

## Introduction

microRNAs (miRNAs) are a class of short non-coding single-stranded molecules with 18–25 nt, involved in the post-transcriptional regulation of gene expression (1, 2). miRNA biogenesis is initiated *via* transcription by RNA polymerase II as part of capped and polyadenylated primary transcripts (pri-miRNAs) (2). The pri-miRNAs are further processed by a complex called “Microprocessor,” consisting of a member of the ribonuclease III family (Drosha) and its cofactor (DGCR8), to release an approximately 65-nt stem-loop precursor miRNA (pre-miRNA). The resulting pre-miRNAs are exported by exportin-5 to the cytoplasm where hairpin precursor sequences are cleaved by the Dicer enzyme to generate 22-bp double-stranded miRNA duplexes. Mature miRNA in the duplexes is released by helicase and assembled into RNA-induced silencing complex (RISC) to recognize and silence the target (3–6).

**Figure 1:**
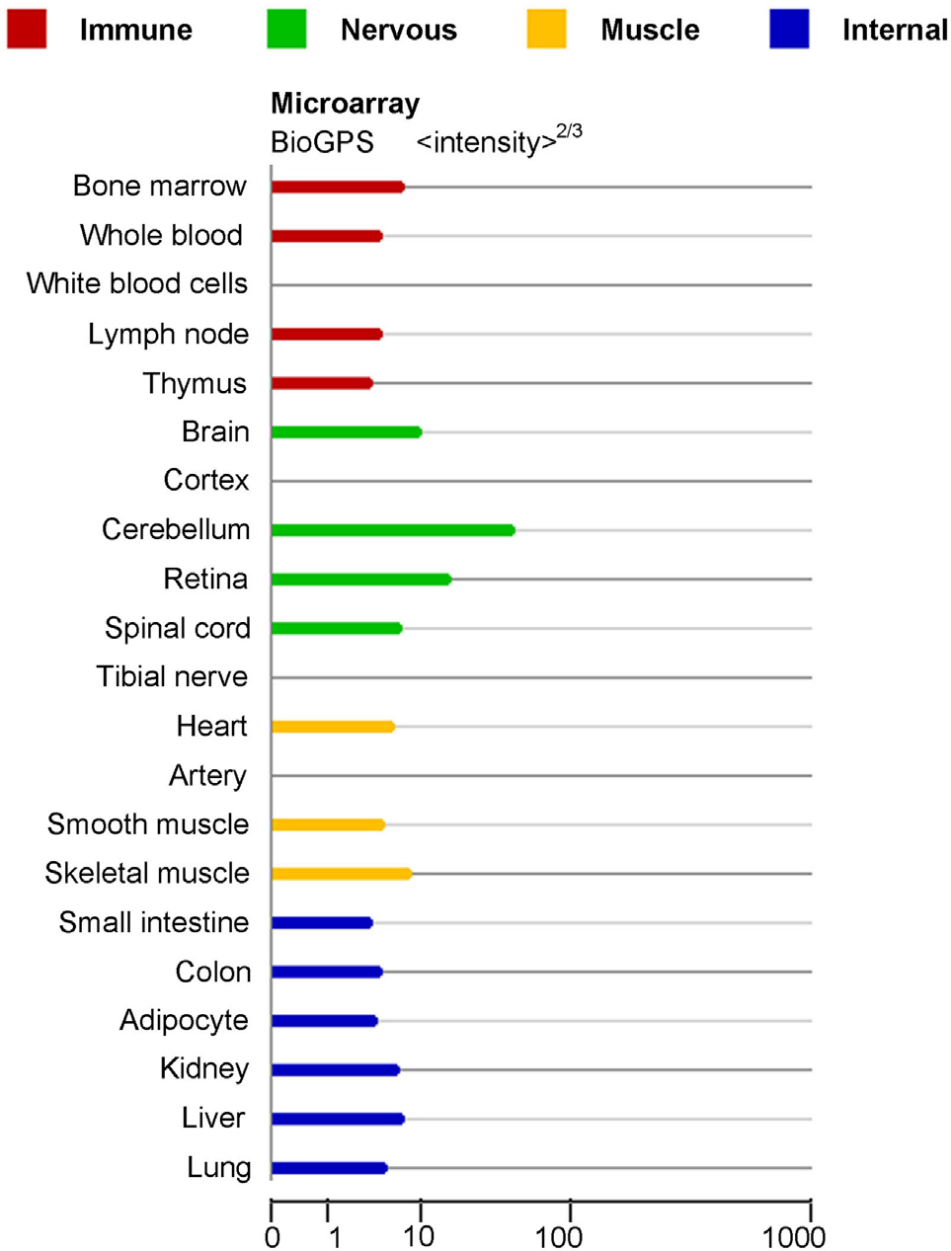
**mRNA expression in normal human tissues for miR-124-1 Gene (http://www.genecards.org/cgi-bin/carddisp.pl?gene=MIR124-1&keywords=miRNA-124)**.

miRNAs are increasingly being distinguished as crucial modulators of gene expression in many biological processes, such as cell maturation (7), differentiation, proliferation (8), metastasis (9), apoptosis (2), and autophagy (10). Specifically, miR-124 was initially verified by cloning studies in mice (11). There are three subtypes of miR-124, named miR-124-1, miR-124-2, and miR-124-3, which have distinct chromosome locations. While the precursors of miR-124 from different species are different, the sequences of mature miR-124 in human, mice, rats are completely identical. Noteworthily, miR-124 is the most abundantly expressed miRNA in neuronal cells and is highly expressed in the immune cells and organs, including peripheral blood mononuclear cells, bone marrow, lymph node, and thymus (Figure 1) (11–13).

miR-124 in the RISC recognizes target mRNAs through imperfect base pairing. Its seed region, approximately 7-nt-long in the 5′-end, mediates its interaction with target mRNAs and determines the target specificity. It usually binds to the 3′-untranslated regions (3′-UTRs) of specific mRNAs to affect their mRNA stability or protein translation (14). Significant progresses have been made in assessing the biological activities and regulation of miR-124, including experimental validation of a number of predicted targets and the unveiling of regulatory mechanism. Thus, it is possible now to comprehend the roles of miR-124 in innate and specific immunity as well as immunity and inflammation-associated diseases.

## miR-124 in the Innate and Adaptive Immune Cells

### miR-124 in Hematopoietic Cells

miRNAs have been demonstrated to regulate hematopoiesis through targeting different genes post-transcriptionally (15). Recently, miR-124 is found to be expressed in a low level in human cord blood CD34(+) cells, but considerably upregulated during culturing and differentiation of these cells. Moreover, miR-124 decreases Tip110 expression and promotes differentiation of human cord blood CD34(+) cells, suggesting an important role of miR-124/Tip110 in hematopoiesis (16).

### miR-124 in the Dendritic Cell Subsets

miR-124 is likely involved in the process of dendritic cell (DC) subset development. DCs include plasmacytoid DCs (pDCs) and classical DCs (cDCs) subpopulations, with the latter consisting of cDC1 and cDC2 lineages. DC subsets from the culture of mouse bone marrow showed high expression of miR-124, which is dominantly expressed in CD24(+) cDC1 cells compared with pDCs and CD172α^+^cDC2 cells. miR-124 is supposed to be involved in the processes of DC subset development by targeting the transcript of TCF4, a critical transcription factor in the development and homeostasis of pDCs (17).

### miR-124 in the Resident Macrophage Polarization

Macrophages function depending on polarization status. M1 macrophages are characterized by a proinflammatory phenotype, while M2 macrophages display anti-inflammatory one (18, 19). It has been reported that resident macrophages nest in various tissues including the central nervous system (CNS), peritoneum, lungs, liver, and adipose tissues. They express a number of M2 markers, such as Ym1, IL-10, IL-4, CD206, Fizz1, and Arg1, suggesting that tissue-resident macrophages are often M2 dominant under normal physiological conditions (20). miR-124 is essential for the induction and maintenance of the M2 phenotype in tissue-resident macrophages, as reflected by miR-124 inhibitor abrogating the upregulation of M2 markers (CD206, Ym1) and downregulation of M1 markers (CD86, iNOS, TNF) in M2-polarized macrophages (21). miR-124 serves as a universal regulator of M2 macrophage polarization, and C/EBP-α is regarded as the mediator of the miR-124 effect on macrophage polarization, although it remains unclear how C/EBP-α suppresses M2 polarization (22).

### miR-124 in the CD4^+^ T Cell Differentiation

miR-124 is a critical mediator for the differentiation of naïve CD4^+^ T cells into T helper type 1 [T(H)1] and T(H)17 cells *in vitro* and *in vivo*, which is regulated by the upstream methyl CpG-binding protein 2 (MeCP2) protein. Loss of MeCP2 in CD4^+^ T cells results in the decreased expression of miR-124, with consequently the accumulation of miR-124-targeting protein suppressor of cytokine signaling 5 (SOCS5). SOCS5 then inhibits STAT1 and STAT3 activation, further represses the differentiation of T(H)1 and T(H)17 cells, respectively (23).

## miR-124 in the Immune Responses

### miR-124 in Innate Immune Responses

The innate immune system coordinates host defenses through pattern recognition receptors, such as toll-like receptors (TLRs) (24). miRNAs represent a component of the innate immune responses that can restrain inflammatory signaling (1, 25, 26). It is reported that miR-124 can be induced upon *Mycobacterium bovis* bacillus Calmette–Guérin (BCG) infection in both RAW264.7 AM cells *in vitro* and murine AMs *in vivo* and is transcriptionally regulated by TLR signaling adaptor MyD88. In turn, elevated miR-124 negatively modulates TLR activity in RAW264.7 cells by directly targeting multiple components of TLR signaling cascade, including the TLR6, myeloid differentiation factor 88 (MyD88), TNFR-associated factor 6 (TRAF6), and TNF-α (27). Moreover, our lab also found activation of TLR4 rapidly increased the level of miR-124 in RAW264.7 macrophages and mice, while miR-124 directly targeted ubiquitin-specific proteases 2 (USP2) and 14 (USP14), two components of deubiquitinating enzymes, to negatively regulate LPS-induced production of TNF-α at the post-transcriptional level (28). Opioids have universal immunosuppressive effects, and the mechanisms are unclear. Qiu et al. have found morphine inhibits the innate immunity in microglia partially through TLRs, and miR-124 mediates this effect. Morphine promotes miR-124 expression in microglia, bone marrow-derived macrophages through upregulating p65 expression, which can directly bind to promoters of miR-124 and enhance miR-124 transcription, where miR-124 mediates morphine’s inhibition of the TLR signaling by directly targeting a subunit of NF-κB p65 and TRAF6 (29).

The cholinergic anti-inflammatory pathway is a type of neurophysiological regulatory mechanism to the immune stimulus, capable of monitoring, and feedback adjusting body inflammatory responses. When meeting inflammatory challenge, the vagus nerve releases acetylcholine, the principal neurotransmitter, to ameliorate the synthesis and release of inflammatory cytokines and guard against tissue damage through the α7 subunit-containing nicotinic acetylcholine receptors (α7nAChRs) in the macrophages. Our lab found that miR-124 is a critical mediator for the cholinergic anti-inflammatory action. miR-124 is upregulated by cholinergic agonists in LPS-exposed cells and mice, and miR-124 further modulates TLR4-induced cytokine production by targeting signal transducer and activator of transcription 3 (STAT3) to decrease IL-6 production and TNF-α converting enzyme (TACE) to reduce TNF-α release (30). These results indicate a negative regulatory role of miR-124 in fine-tuning the TLR-triggered inflammatory responses.

### miR-124 in Adaptive Immune Responses

T lymphocytes are the main executors of the adaptive immune responses. miR-124 is reported to exert potent anti-glioma therapeutic effects *via* a T cell-mediated antitumor immune response. Glioma cancer stem cells (gCSCs) produce immunosuppressive roles in the T-cell proliferation, and miR-124 could reverse this suppressive effect and decrease the generation of Foxp3^+^ regulatory T-cells (Tregs). Furthermore, treatment of T-cells with miR-124 induces marked effector response including the upregulation of IL-2, IFN-γ, and TNF-α. STAT3, a key pathway mediating immunosuppression in the tumor microenvironment, is identified as the target of miR-124 in the regulation of T-cell functions. In addition, miR-124 also modulates T-helper cell differentiation. When miR-124 is overexpressed, IL-17A + T_H_17 cells and FoxP3^+^ Treg induction is inhibited, whereas IFN-γ + T_H_1 cells differentiation is strengthened (31).

miR-124 also mediates the effect of IL-7 in the generation and maintenance of memory T cells and benefits survival and expansion of HIV-1-latently infected memory CD4^+^ T lymphocytes. IL-7 could elevate miR-124 level, which targets and downregulates polypyrimidine tract-binding protein (PTB) and subsequently leads to the elevation of CD95 on memory CD4^+^ T cells. Elevated CD95, combined with coactivated FLICE-like inhibitory protein (c-FLIP) and JNK by IL-7, primes CD4^+^ T lymphocytes to a survival mode and contributes to the IL-7-mediated maintenance of HIV-1 reservoir (32).

Critical illness-related corticosteroid insufficiency (CIRCI) is defined as inadequate corticosteroid activity for the severity of the illness. Sepsis is often accompanied with acquired glucocorticoid resistance, which complicates the therapy of sepsis. T-cell system is the key player in the adaptive immune response and is considered as one of the key players in the pathogenesis of CIRCI. Recent studies showed that miR-124 mediates the glucocorticoid resistance in human T-cells. T-cells from sepsis patients exhibit decreased glucocorticoid receptor-α (mediating anti-inflammatory effects) and increased miR-124 levels. Further studies demonstrated that glucocorticoid significantly elevates miR-124 level, which specifically downregulates glucocorticoid receptor-α and limits anti-inflammatory effects of glucocorticoids (33).

## miR-124 in Immune Diseases

Many inflammatory diseases and immune disorders witness altered miR-124 expression. Further characterization of these regulatory networks triggers mechanistic studies. The involvement of miR-124 in immunity-related diseases and their identified targets are discussed in detail as followed.

### miR-124 in CNS Immune-Related Diseases

miR-124 is one type of brain-specific miRNAs and has been revealed to mediate the function of immune system in the CNS (12, 34). miR-124 was first identified as a key regulator of promoting microglia, one kind of macrophages that are resident in the brain and spinal cord, quiescence. miR-124 expression is decreased in activated microglia during the course of experimental autoimmune encephalomyelitis (EAE), while overexpression of miR-124 could promote activated microglia into a phenotype resembling resting microglia and suppress EAE by deactivating macrophages *via* the C/EBP-α-PU.1 pathway (22). Spinal cord injury remains a difficulty to be treated due to the secondary inflammatory damage induced by activated microglia and/or macrophages. Louw et al. found that miR-124 transfection decreased the activation of rat microglia. Microinjected particles of chitosan/miR-124 in the peritoneum, which will be transported by macrophages to the site of spinal cord injury 72 h post injection, decrease the infiltration of macrophages in the injured spinal cord, and therefore ameliorate spinal cord injury (35). Chronic pain is often associated with microglia activation in the spinal cord. Willemen et al. have found that persistent hyperalgesia in LysM-GRK2^+/−^ mice is associated with reduced miR-124 levels in spinal cord microglia, while intrathecal miR-124 treatment can be used to prevent and treat persistent inflammatory and neuropathic pain by restoring the increased ratio of M1/M2 type markers in spinal cord microglia/macrophages (36).

However, miR-124 does not always act as a repressor for microglia activation. Epilepsy is closely relevant to dysregulated inflammatory pathways. Brennan et al. have proved that status epilepticus leads to suppressed miR-124 expression, while synthetic miR-124 significantly augments microglia activation and inflammatory cytokines. Further studies have demonstrated that miR-124 acts as a dual regulator in epilepsy, which attenuates epileptogenesis *via* targeting NRSF while promoting epilepsy *via* exaggerating inflammation (37).

### miR-124 in Peripheral Immune-Related Diseases

Inflammatory bowel diseases (IBDs), which refer to ulcerative colitis (UC) and Crohn’s disease (CD), are multifactorial diseases with probable genetic heterogeneity. Environmental risk factors, such as diet, smoking, measles, or appendectomy, may contribute to their pathogenesis. The mechanism of IBDs has only been partly understood. Recent reports indicated that miR-124 might be involved in their regulation. Koukos et al. found that miR-124 is downregulated in tissues from children with UC due to promoter hypermethylation, while reduced levels of miR-124 promote inflammation and the pathogenesis of UC by increasing expression and activity of STAT3 in colon tissues. Interestingly, the same group did not observe the decrease of miR-124 in adult UC tissues, reflecting the mechanistic differences between adults and pediatric UC (38). Inversely, active CD patients were found to have higher level of miR-124 in colon tissues and intestinal epithelial cells (IECs). miR-124 can directly target AHR protein to modulate proinflammatory cytokine production and thereby promote the pathogenesis of CD (39, 40). As we know, smoking plays a dual role in IBD by decreasing the risk of UC and increasing that of CD (41), the potential mechanisms are still unknown. It has been reported that nicotine is thought to be the most important agent responsible for the effect of smoking, and the dual role of miR-124 in the treatment of UC and CD may be responsible for this discrepancy, which needs to be further explored.

Rheumatoid arthritis (RA) is an autoimmune disease in which the body’s immune system mistakenly attacks the joints. It is recently reported that RA patients have higher miR-124 promoter methylation, while healthy controls have not such a modification, suggesting that epigenetic dysregulation may be involved in the development of RA (42). Moreover, Nakamachi et al. found that miR-124 level significantly decreased in RA synoviocytes as compared with osteoarthritis (OA) synoviocytes, and miR-124 could target cyclin-dependent kinase 2 (CDK2) or monocyte chemotactic protein 1 (MCP1), adjusting proliferation and the ability to produce chemokines of fibroblast-like synovial cells (43). Subsequently, they also found that forced expression of miR-124 repressed adjuvant-induced arthritis (AIA) in rats by suppressing RANKL and NFATc1, resulting in the decreased synoviocyte proliferation, leukocyte infiltration, and cartilage or bone destruction (44). Thus, miR-124 is a promising therapeutic target in human RA.

Ankylosing spondylitis (AS) is a type of arthritis that affects the spine, and symptoms include pain and stiffness from the neck down to the lower back. The spine’s bones (vertebrae) may grow or fuse together, resulting in a rigid spine. miR-124 is upregulated and ANTXR2 is downregulated in peripheral blood from AS patients. Further studies demonstrated that miR-124 targeted ANTXR2 and inhibited its expression in Jurkat cells, leading to the activation of JNK and inducing autophagy. miR-124-ANTXR2 might be a therapeutic target for AS (45).

### miR-124 in Tumor Immunity

Many studies have demonstrated the potential application of miR-124 as a novel immunotherapeutic agent for tumors, and several targets have been identified. For example, miR-124 can inhibit tumor by directly targeting STAT3, a key component mediating immunosuppression in the tumor microenvironment. Bellon et al. found miR-124 inhibited adult T-cell leukemia (ATL) cell proliferation *in vitro* and tumor growth *in vivo* of ATL mouse model by targeting STAT3-Pim1 pathway (46). Wei et al. reported that systemic administration of miR-124 or adoptive miR-124-transfected T-cell-suppressed glioma by repressing the STAT3 pathway (31). miR-124 can suppress the fitness of B-cell lymphomas by targeting MYC and BCL2, which are coexpressed in B-cell lymphomas and associated with the poor prognosis (47). Meanwhile, miR-124 also increases sensitivity of B-cell lymphomas to glucocorticoid treatment by targeting phosphodiesterase 4B (PDE4B) and may act as an attractive therapeutic target in B cell lymphoma (48).

miR-124 can exert tumor suppressive effects in human liver cancers. Hepatocyte nuclear factor 4α (HNF4α) reduction contributes to liver tumorigenesis and inflammation. Hatziapostolou et al. reported that miR-124 is an integral part of the circuit and a direct downstream effector of HNF4α activity, targeting IL-6R, and modulating IL-6R/STAT3 pathway during hepatocellular transformation. Systemic administration of miR-124 suppresses hepatocellular carcinogenesis by inducing tumor-specific apoptosis (49). Coincidentally, Ning et al. have reported that miR-124 also participates in HNF4α–NF-κB feedback circuit in hepatocellular carcinoma. miR-124 is elevated by HNF4α transcriptionally, inhibiting RelA (p65) expression *via* interaction with RelA–3′-UTR (50). Intriguingly, an activated NF-κB-centered inflammatory loop was also detected in node-positive non-small cell lung carcinoma (NSCLC). NF-κB-regulated miR-124 targets MYO10, inhibiting cell invasion and metastasis (51).

Particularly, miR-124 is known to be methylation silenced during exposure to chronic inflammation, which is correlated with the higher epidemiologic risk of cancer. Ueda et al. have reported that three miR-124 genes are methylated, whereas CDK6 is highly expressed in tissues from patients with colitis-associated cancer (CAC), sporadic colorectal cancer, and dysplasia. During carcinogenesis in UC patients, miR-124 genes are methylated. The methylation level of miR-124-3 could be recognized as a valuable marker for estimating individual risk of CAC (52). Methylation of hsa-miR-124-2 was previously found to be indicative for cervical (pre)cancer. Methylation markers combined with HPV testing might offer a full molecular screening strategy to the many HIV-infected women who are also hrHPV-positive (53).

### miR-124 in Viral Immunity

Several studies have revealed alterations in cellular miRNA profiles following HIV-1 infection, which are mostly involved in inhibiting viral infection. However, several miRNAs, including miR-124, were found to modulate viral spread in T-lymphocytes and HeLa-CCR5 cell lines. Following infection, let-7c, miR-34a, or miR-124 was upregulated, and they targeted and downregulated p21 and TASK1, which eventually led to increased virion release and higher copy number of viral genome transcripts in infected cells, suggesting that HIV-1 could utilize the host miRNA cellular systems to produce a more efficient infection process *via* blocking the mechanism of innate inhibition (54). Additional studies have demonstrated this regulation. Through inducing miR-124, IL-7 has been shown to elevate CD95 in CD4(+) T cells from HIV-1-infected individuals and prime CD4(+) T lymphocytes to CD95-mediated signals, leading to the IL-7-mediated maintenance of HIV-1 reservoir (32).

miRNA deep sequencing was performed to probe into the contribution of miRNAs to the host immune response to virus infection. Intriguingly, pathway analysis of miRNAs and targets has shown that increased miR-124-3p interplays with innate immunity-related pathways in spleen tissues of mice infected with A/Swine/GD/2/12 (H1N1) virus. Further master of the roles played by these miRNAs in influenza virus infection will acquire a good comprehension of host–pathogen interactions (55).

Bian et al. found miR-124 specifically responded to the Enterovirus 71 (EV71) infection and interferon (IFN) treatment, and they further predicted some related signaling pathways, which would be helpful to elucidate the interaction between the virus and the host (56). In addition, the expression of hsa miR-124-3p is significantly upregulated in the HCMV latent infection library (57). Japanese encephalitis virus (JEV) can infect neurons and directly cause lethal encephalitis. Yen et al. inserted two copies of a perfectly matched miR-124 recognition element into the 3′-UTR of viral RNA to create infectious JEV recombinant RP-124PT (rRP-124PT). The effect of rRP-124PT was attenuated in infected mice as compared with MRE mutant and parental strains. Immunization with rRP-124PT appeared to elicit full protective immunity against subsequent JEV lethal challenge, indicating that endogenous neuron-specific miR-124 could be used to restrict viral neurotropism and consequently diminish the neurovirulence of JEV in mice (58).

## Conclusion and Perspectives

Large numbers of studies in different cells and systems have found that miR-124 is a critical modulator of immune cells, inflammatory, and immunological responses. In many cases, miR-124 is induced by the inflammatory or immunological signals and, in turn, functions as a negative regulator for these signals, forming a negative feedback to help maintain homeostasis. Physiological and pathological changes and their significance of miR-124 are summarized in Table 1. Noteworthily, it has been demonstrated that a particular miRNA could often target multiple proteins, and a particular protein might be regulated by multiple miRNAs. Therefore, algorithm analysis based on miRNA profile as well as molecules in activated signal pathway will likely provide a more comprehensive picture for understanding the regulatory action of miRNA (56). Such analysis may also shed new insight on the roles of miR-124 in infectious and immunological diseases.

**Table 1:** **miR-124 in immune system and immune disorders**.

Physiological and pathological conditions	Expression	Target	Action	Reference
Hematopoiesis differentiation	Upregulation in the differentiation of human cord blood CD34(+) cells	Tip110	Regulate hematopoiesis differentiation	(16)
DC subset development	High expression in CD24(+) cDC1 cells during the culture of mouse bone marrow	TCF4	Regulate DC subset development	(17)
Resident macrophage polarization	Constitutive expression in macrophages	–	Promote M2 polarization of resident macrophages	(21)
Experimental autoimmune encephalomyelitis (EAE)	Downregulation in activated microglia	C/EBP-α-PU.1	Promote microglia quiescence and protect against EAE	(22)
CD4^+^ T cell differentiation	Upregulation in the process of CD4^+^ T cell differentiation	SOCS5	Stimulate naïve CD4^+^ T cells to differentiate into T helper type 1 [T(H)1] and T(H)17 cells	(23)
BCG infection	Upregulation in alveolar macrophages upon BCG infection	TLR6, MyD88, TRAF6, and TNF-α	Restrain inflammatory signaling	(27)
TLR4 signaling	Upregulation in LPS-stimulated RAW264.7 macrophages and mice	USP2, USP14	Negatively regulate TNF-α production	(28)
Morphine’s effect in innate immunity	Upregulation in microglia and macrophages treated by morphine	NF-κB p65 and TRAF6	Mediate morphine’s inhibition in the TLR signaling	(29)
Cholinergic anti-inflammation pathway	Upregulation by cholinergic agonists	STAT3 and TACE	Critical mediator for the cholinergic anti-inflammatory action	(30)
Gliomas	Absence in all grades and pathological types of gliomas	STAT3 in T cells and in glioma cancer stem cells (gCSC)	Anti-glioma effects	(31)
Corticosteroid resistance in sepsis	Upregulation by glucocorticoid in T cells	GR-α	Limit anti-inflammatory effects of glucocorticoids	(33)
Spinal cord injury	Forced expression	–	Decrease microglia activation and ameliorate spinal cord injury	(35)
Chronic pain	Downregulation in spinal cord microglia	–	Increase the ratio of M1/M2 type markers, and prevent or treat chronic pain	(36)
Status epilepticus	Downregulation	Inhibit NRSF and augment microglia activation	Attenuate epileptogenesis *via* targeting NRSF while promote epilepsy *via* exaggerating inflammation	(37)
Ulcerative colitis (UC)	Downregulation in colon tissues from children with UC	STAT3	Inhibit inflammation and the pathogenesis of UC	(38)
Crohn’s disease (CD)	Upregulation in colon tissues and intestinal epithelial cells	AHR	Modulate proinflammatory cytokine production and promote the pathogenesis of CD	(40)
Rheumatoid arthritis (RA)	Downregulation in RA synoviocytes	CDK-2, MCP-1, NFATc1, and RANKL	Decrease synoviocyte proliferation, leukocyte infiltration and cartilage or bone destruction, and protect against RA	(43, 44)
Ankylosing spondylitis (AS)	Upregulation in peripheral blood from AS patients	ANTXR2	Therapeutic action for AS	(45)
Adult T-cell leukemia (ATL)	Downregulation	STAT3-Pim1	Inhibit ATL-cell proliferation and tumor growth of ATL mouse model	(46)
B-cell lymphomas (BCL)	Downregulation	P65	Suppress the fitness of BCL	(47)
		MYC		
		BCL2		
		PDE4B	Increase sensitivity of BCL to glucocorticoid	(48)
Liver cancers	Downregulation in hepatocellular oncogenesis	IL-6R and P65	Tumor suppressive effects	(49, 50)
Non-small cell lung carcinoma (NSCLC)	Downregulation in highly invasive sub-cell lines and node-positive NSCLC specimens	MYO10	Inhibit cell invasion and metastasis	(51)
Colitis-associated cancer (CAC)	Methylation in tissues from patients with CAC	CDK6	The methylation level of miR-124-3 is a promising marker for estimating individual risk of CAC	(52)
Cervical (pre)cancer	Methylation	–	Indication of cervical (pre)cancer	(53)
HIV-1 infection	Upregulation following infection	p21 and TASK1	Increase viral spread in T-lymphocytes and HeLa-CCR5 cell lines	(54)
	Upregulation by IL-7	PTB	Benefit survival and expansion of HIV-1-latently infected memory CD4^+^ T lymphocytes	(32)
A/Swine/GD/2/12 (H1N1) virus infection	Upregulation	Innate immunity-related pathways	Pathway analysis	(55)
Enterovirus 71 (EV71) infection	Upregulation	–	Indicate virus and the host interaction	(56)
HCMV latent infection	Upregulation of hsa miR-124-3p	–	–	(57)

The alterations of miR-124 have been found in many inflammatory and immunological diseases. Therefore, assessing miR-124 in various body fluids (59–61) might be an informative biomarker for the timely diagnosis and prognosis of inflammatory diseases. Additionally, stable miR-124 mimic and inhibitor are candidates for therapeutics that modulate inflammatory and immunological diseases. Thus, further exploring the specific roles of miR-124 under physiological and pathological conditions may likely bear fruitful results in the foreseeable future.

Recently, a report has aroused widespread attention that precursor miRNAs could compete with their mature miRNA counterparts. Roy-Chaudhuri et al. validated this for miR-124 and the SNAI2 3′-UTR. Therefore, miR-124 precursors are expected to serve as post-transcriptional regulators of miRNA activities rather than mere biogenesis midbodies (62). The regulatory role of miR-124 precursors in immune regulation might be a novel research frontier.

## Author Contributions

ZQ and P-YW retrieved and analyzed concerned literatures. ZQ wrote the manuscript. XL and D-FS revised the manuscript. All authors agree to be accountable for the content of the work.

## Conflict of Interest Statement

The authors declare that the research was conducted in the absence of any commercial or financial relationships that could be construed as a potential conflict of interest.
